# The Role of Swelling in the Regulation of OPA1-Mediated Mitochondrial Function in the Heart In Vitro

**DOI:** 10.3390/cells12162017

**Published:** 2023-08-08

**Authors:** Xavier R. Chapa-Dubocq, Keishla M. Rodríguez-Graciani, Jorge García-Báez, Alyssa Vadovsky, Jason N. Bazil, Sabzali Javadov

**Affiliations:** 1 Department of Physiology, University of Puerto Rico School of Medicine, San Juan, PR 00936-5067, USA; xavier.chapa@upr.edu (X.R.C.-D.); keishla.rodriguez20@upr.edu (K.M.R.-G.); jorge.garcia25@upr.edu (J.G.-B.); 2 Department of Physiology, Michigan State University, East Lansing, MI 48824-1046, USA; vadovsky@msu.edu (A.V.); jnbazil@msu.edu (J.N.B.)

**Keywords:** heart mitochondria, OPA1, mitochondrial swelling, membrane potential, calcium retention capacity, mitochondrial respiration

## Abstract

Optic atrophy-1 (OPA1) plays a crucial role in the regulation of mitochondria fusion and participates in maintaining the structural integrity of mitochondrial cristae. Here we elucidate the role of OPA1 cleavage induced by calcium swelling in the presence of Myls22 (an OPA1 GTPase activity inhibitor) and TPEN (an OMA1 inhibitor). The rate of ADP-stimulated respiration was found diminished by both inhibitors, and they did not prevent Ca^2+^-induced mitochondrial respiratory dysfunction, membrane depolarization, or swelling. L-OPA1 cleavage was stimulated at state 3 respiration; therefore, our data suggest that L-OPA1 cleavage produces S-OPA1 to maintain mitochondrial bioenergetics in response to stress.

## 1. Introduction

Mitochondria are intracellular organelles which consist of two membranes known as the inner mitochondrial membrane (IMM) and the outer mitochondrial membrane (OMM); these together form the intermembrane space between them as well as the matrix. The IMM is subdivided into two morphologically different domains known as the inner boundary membrane (the IMM regions in proximity to the OMM) and the cristae membrane (the invaginated regions of the IMM). Cristae morphology dictates mitochondrial respiratory capacity, since the cristae are the main IMM regions responsible for energy conversion [1,2]. The cristae membrane contains an abundance of proteins such as the ETC complexes, F_O_F_1_-ATP synthase, optic atrophy 1 (OPA1) and the mitochondrial contact site and cristae organizing system (MICOS) that regulate mitochondrial function. In response to various physiological stimuli, mitochondria undergo cristae remodeling to maintain their functional stability. Under stress conditions associated with increased reactive oxygen species and Ca^2+^ overload, the MICOS multicomplex and OPA1 can be disrupted, which would impair the structural integrity of the cristae junctions and ultimately result in mitochondrial respiratory defects.

OPA1 is a mitochondrial dynamin-like GTPase which, in addition to the fusion of mitochondria, has been shown to play a key role in the maintenance and stability of the cristae structure [2]. In humans, OPA1 exists as eight different isoforms which can be expressed as combinations of long (L) and short (S) forms known as L-OPA1 and S-OPA1, respectively [3]. The different isoforms of OPA1 can be oligomerized to maintain tight cristae junctions and thereby prevent cytochrome c release from the intercristal space [4]. In addition, through interaction with cardiolipin, the main phospholipid of the mitochondria, L-OPA1 acts as a contact site to tether with the L-OPA1 of another mitochondrion, thereby facilitating IMM fusion [5]. Regulating mitochondrial fusion and the formation of cristae junctions is important for mitochondrial quality control and cell viability.

YME1L and OMA1 are the primary enzymes involved in the proteolytic cleavage of L-OPA1. Under physiological conditions, L-OPA1 is cleaved by the ATP-dependent metalloprotease YME1L to form S-OPA1. However, under stress conditions accompanied by membrane depolarization and ATP depletion, YME1L is rapidly degraded, and the ATP-independent zinc metalloprotease OMA1 is activated and becomes the primary enzyme for L-OPA1 cleavage [6]. L-OPA1 is cleaved at different amino acid sequence sites; OMA1 cleaves at the S1 site whereas YME1L cleaves at the S2 site [7]. L-OPA1 has been demonstrated to be the main component in stabilizing the cristae structure in comparison to S-OPA1, which has a less significant stabilizing role [8,9]. Several studies have shown that increased S-OPA1 levels due to L-OPA1 cleavage are associated with altered metabolic activity of mitochondria [10,11,12,13]. However, recent genetic studies have found that S-OPA1 can maintain both mitochondrial cristae structure and respiratory activity, even when the mitochondria lack fusion capacity [9,14]. Currently, the role of S-OPA1 in the structural organization of mitochondrial cristae and regulation of mitochondrial function remains unclear.

We have previously shown that Ca^2+^-induced mitochondrial swelling stimulates proteolytic cleavage of L-OPA1 in isolated cardiac mitochondria [15]. This would suggest that maintaining L-OPA1 integrity may be beneficial under stress conditions such as Ca^2+^-induced mitochondrial swelling. Myls22, a pharmacological compound, serves as an inhibitor of the GTPase activity exhibited by both L-OPA1 and S-OPA1. By virtue of its inhibitory properties, Myls22 effectively impedes mitochondrial fusion activity. Recent studies have demonstrated that the inhibition of OPA1 GTPase activity by Myls22 is protective against breast cancer growth [16]. Similarly, inhibition of OMA1 by N,N,N’,N’-tetrakis-(2-pyridylmethyl)ethylenediamine (TPEN) reduced mitochondrial damage induced by optic nerve injury in C57BL/6J mice [17], suggesting beneficial effects of L-OPA1 under stress conditions. It is important to mention, however, that TPEN’s ability to chelate zinc may have unspecific impacts on mitochondria. In this study, we sought to clarify the relationship between L-OPA1 cleavage and the respiratory function of mitochondria using pharmacological inhibitors rather than genetic silencing. We showed that L-OPA1 cleavage was favorable under ADP-stimulated respiration (state 3), suggesting a potential role of S-OPA1 in mitochondrial bioenergetics. This study also demonstrates that inhibition of both OPA1 and OMA1 is ineffective in preventing mitochondrial dysfunction at high Ca^2+^; therefore, the role of OPA1 for maintaining IMM structural integrity and function may be limited under pathological conditions.

## 2. Materials and Methods

### 2.1. Animals

Male Sprague Dawley rats (275–325 g) were purchased from Taconic (Hillside, NJ, USA). All the experiments were performed according to protocols approved by the UPR Medical Sciences Campus Institutional Animal Care and Use Committee and conformed to the National Research Council Guide for the Care and Use of Laboratory Animals published by the US National Institutes of Health (2011, eighth edition).

### 2.2. Cardiac Mitochondria Isolation

The isolation of mitochondria was adopted and modified from previous studies [18]. Briefly, both heart ventricles were cut and homogenized using a Polytron homogenizer in 20 mL ice-cold sucrose buffer containing (in mM): 300 sucrose, 20 Tris-HCl, and 2 EGTA, pH 7.2, and supplemented with 0.05% fatty acid-free BSA. The heart homogenate was centrifuged at 2000× *g* for 3 min to remove cell debris. The supernatant was centrifuged at 10,000× *g* for 6 min to precipitate the mitochondria in the sucrose buffer (BSA-free) and then washed again under the same conditions in a swelling assay buffer to reduce the EGTA concentration. The final pellet containing the mitochondria was resuspended in 300 µL of the swelling assay buffer with a final concentration of ~10–15 µg/µL. The swelling assay buffer contained (in mM): 125 KCl, 20 MOPS, 10 Tris-HCl, 2 MgCl_2_, 0.001 EGTA, and 2 KH_2_PO_4_, pH 7.1.

### 2.3. Analysis of mPTP Opening

The swelling of the mitochondria in the presence or absence of Ca^2+^ was determined using freshly isolated mitochondria (50 μg) by monitoring the decrease in light scattering at 525 nm as previously described, with minor modifications [18]. The experiments were performed on a CLARIOstar microplate reader (BMG Labtech, Cary, NC, USA) using a 96-well plate. The swelling curves were averaged and presented as their mean absorbance value. The experiments were performed at 37 °C in 0.1 mL of swelling assay buffer or in a hypotonic buffer containing (in mM): 25 KCl, 20 MOPS, 10 Tris-HCl, 2 MgCl_2_, 0.001 EGTA, and 2 KH_2_PO_4_, pH 7.1. The pore-forming agent alamethicin was used to induce complete mitochondrial swelling [19].

### 2.4. Calcium Retention Capacity (CRC) Assay

The CRC was measured by the Ca^2+^-sensitive fluorescence dye Fluo-5N which reacts to extramitochondrial Ca^2+^ in the assay buffer [20]. Briefly, freshly isolated mitochondria (50 μg or 0.5 mg/mL) were incubated at 37 °C in 0.1 mL of swelling assay buffer containing 500 nM of Fluo-5N. Exogenous Ca^2+^ was added to increase the matrix Ca^2+^ load, and the fluorescence intensity was recorded by means of a CLARIOstar microplate reader (BMG Labtech, Cary NC, USA) using a 96-well plate. The CRC curves were averaged and presented as fluorescence intensity (relative fluorescent units).

### 2.5. Analysis of Mitochondrial Respiration and Membrane Potential

Measurement of mitochondrial respiration and membrane potential was performed at 37 °C using an Oxygraph 2k (Oroboros Instruments Corp., Innsbruck, Austria). The O2k chambers were loaded with 2 mL of a swelling assay buffer that contained (in mM): 125 KCl, 20 MOPS, 10 Tris-HCl, 2 MgCl_2_, 0.001 EGTA, and 2 KH_2_PO_4_, pH 7.1. All the subsequent experiments were performed using this buffer and temperature. At 0 min, the inhibitors (as indicated in figures) 2.5 mM 2-oxoglutarate and 1 mM L-malate (OM) as well as 0.1 μM of the lipophilic cationic dye TMRM were added, followed by 0.1 mg/mL of mitochondria. Here, we considered state 2 respiration as the rate of oxygen consumption by the mitochondria only in the presence of substrates, whereas state 3 was performed in the presence of 1 mM ADP in addition to the substrates. At 5 min, a 600 nmol/mL bolus of Ca^2+^ was added to induce mitochondrial swelling. At 10 min, a 1 mM bolus of ADP was added to induce maximal ADP-stimulated (state 3) respiration. An alternative experiment was performed in which we assessed the same parameters with ADP added at 0 min and the Ca^2+^ bolus at 5 min. The ratiometric approach (546/573 nm excitation, 590 nm emission) was applied to calculate the membrane potential on the basis of the TMRM fluorescence activity by the following equation: $\frac{F(t=0)-F(t)}{F_{max}-F_{min}}$ [21]. This equation is utilized to evaluate changes in fluorescence intensity and allows for the normalization of the fluorescence signal. Several factors, such as fluorescent molecule concentration, excitation intensity, and instrument variations, can influence fluorescence intensity. However, the equation enables researchers to determine the relative change in fluorescence intensity independent of these factors, thereby providing a direct correlation to the biological process under scrutiny.

### 2.6. SDS-PAGE and Western Blotting

Equal numbers of mitochondrial proteins were resolved by SDS-PAGE and transferred overnight to nitrocellulose membranes (GE Healthcare Bio-Sciences, Piscataway, NJ, USA). Then the membranes were immunoblotted with OPA1 (#612607, BD Biosciences, Franklin Lakes, NJ, USA,) and ATP5A (#ab14748, Abcam, Boston, MA, USA,). Images were acquired using the Odyssey CLx Infrared Imaging System (LI-COR Biosciences, Lincoln, NE, USA). Image analysis was performed using ImageJ (version 1.52a) software from NIH. The L-OPA1, and S-OPA1 levels were quantified as a percentage of the total OPA1.

### 2.7. Statistical Analysis

The data values for the bar graphs are presented as mean ± SE, whereas time series plots are presented by only the mean. The number of biological samples, but not technical replicates, were used as a sample size. The data were analyzed using the one-way ANOVA and Student’s t-tests for the comparison of independent groups via a GraphPad prism (version 9).

## 3. Results

### 3.1. The Effects of Myls22 and TPEN on Mitochondrial CRC

**Figure 1:**
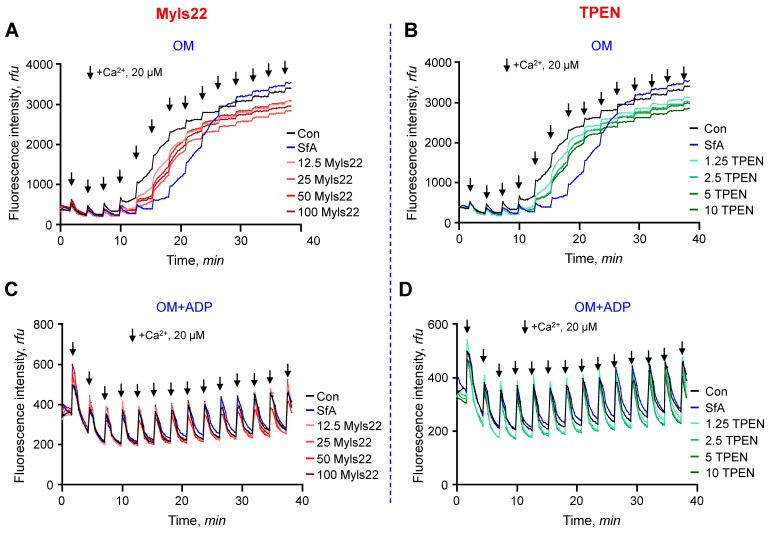
Dose-dependent effects of Myls22 and TPEN on the CRC of isolated cardiac mitochondria. Dose-dependent experiments were conducted using Myls22 (OPA1 inhibitor) and TPEN (OMA1 inhibitor) to determine the effective concentration of each inhibitor. The CRC of mitochondria (0.5 mg/mL) was analyzed in the presence of OM alone (OM group) or in combination with ADP (OM+ADP group). Ca^2+^ was added every 3 min, indicated by arrows, with an incremental increase of 20 µM per pulse. (**A**,**B**) OM group, (**C**,**D**) OM+ADP group. Concentration ranges for Myls22 (**A**,**C**) and TPEN (**B**,**D**) were 12.5–100 µM and 1.25–10 µM, respectively. n = 3 per group.

In the first set of experiments, we evaluated the effects of Myls22 and TPEN on the CRC of isolated heart mitochondria. Considering that L-OPA1, but not S-OPA1, is the main component in stabilizing the cristae structure, we proposed that inhibition of L-OPA1 cleavage through blocking OMA1 activity by TPEN could maintain the IMM structural integrity, increase its resistance to swelling, and enhance the CRC of the mitochondria in response to Ca^2+^. S-OPA1 generated by L-OPA1 cleavage has been shown to retain the GTPase activity and contain all of the functional domains of OPA1 [22]. It should be noted that the zinc-chelating capacity of TPEN may have nonspecific effects on mitochondria. On the other hand, Myls22 can inhibit the GTPase activity of both L-OPA1 and S-OPA1, which is used to prevent fusion activity, thereby limiting the OPA1 function to maintaining the cristae structure [16]. Mitochondria suspensions (50 µg per well) were incubated with Myls22 and TPEN at a range of 12.5–100 µM and 1.25–10 µM, respectively, in the presence of complex I substrates alone (OM group) or in combination with ADP (OM+ADP group). ADP was added to assess the contribution of mitochondrial permeability pores (mPTP)-independent and mPTP-dependent swelling. The concentration ranges for Myls22 and TPEN were based on previous studies [23,24]. The results showed that in the presence of OM, Myls22 was effective at all concentrations (Figure 1A), whereas TPEN was most effective at 2.5–10 µM (Figure 1B) to increase the CRC of the mitochondria by reducing the rate of Ca^2+^ release. The effects of Myls22 and TPEN on the CRC were negligible in comparison to 1 µM sanglifehrin A (SfA, a mPTP inhibitor, positive control). In the presence of OM and ADP, Myls22 and TPEN at all concentration ranges had no noticeable effect on the CRC (Figure 1C,D). Based on concentration-dependent data, 50 µM Myls22 and 5 µM TPEN were used in the next sets of experiments.

### 3.2. The Effects of Myls22 and TPEN on Mitochondrial Function

**Figure 2:**
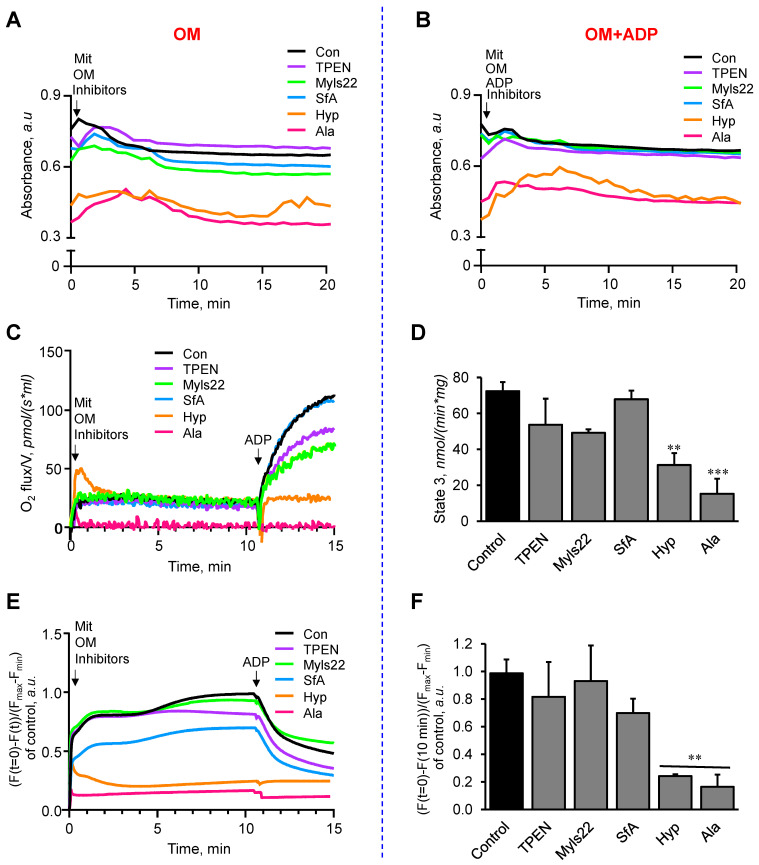
The effects of Myls22 and TPEN on mitochondrial swelling, membrane potential, and respiration. Mitochondrial swelling (**A**,**B**), mitochondrial respiration (**C**,**D**), and membrane potential (**E**,**F**) were analyzed in the presence and absence of ADP. Mitochondrial swelling was measured by TMRM separately in the OM (**A**) and OM+ADP (**B**) groups, whereas mitochondrial respiration (**C**,**D**) and membrane potential (**E**,**F**) were determined simultaneously by adding ADP following basal conditions. All three parameters were measured in the presence of Myls22 (50 µM), TPEN (5 µM), SfA (0.5 µM), hypotonic medium (Hyp), and alamethicin (Ala, 10 µM). n = 3 per group. ** *p* < 0.01 and *** *p* < 0.001 vs. Con.

In these experiments, we examined the effects of Myls22 and TPEN on mitochondria by assessing mitochondrial swelling, respiration, and membrane potential. We found that Myls22 and TPEN did not have any negative effects on mitochondrial swelling in the presence of OM (OM group) or OM and ADP (OM+ADP group) (Figure 2A,B). Likewise, mitochondrial respiration rates (state 2 and state 3) (Figure 2C,D) and membrane potential (Figure 2E,F) were not affected by Myls22 and TPEN; however, they were significantly reduced by alamethicin and hypotonic conditions.

**Figure 3:**
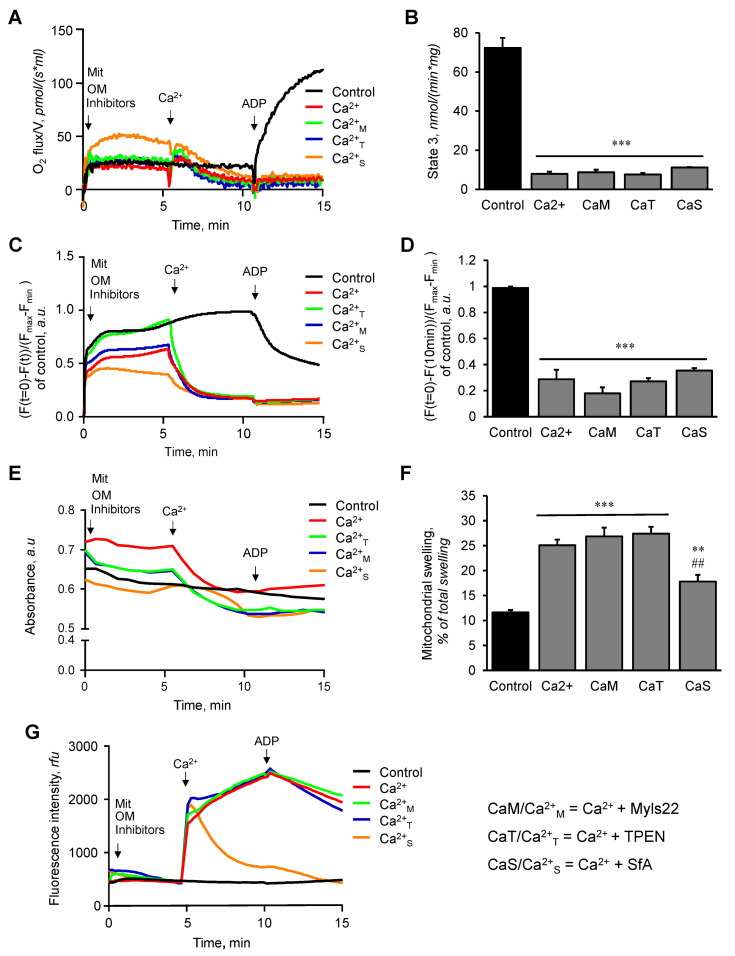
The effects of Myls22 and TPEN on the functional state of mitochondria in the presence of Ca^2+^. The effects of Myls22 and TPEN on mitochondrial respiration (**A**,**B**), membrane potential (**C**,**D**), swelling (**E**,**F**), and CRC (**G**) were analyzed in the presence of Ca^2+^ (600 nmol/mL) and respiration substrates (OM). Quantitative data are presented for state 3 respiration rate (**B**) at 15 min and for membrane potential (**D**) and mitochondrial swelling (**F**) at 10 min after addition of Ca^2+^ and ADP. The inhibitors OM and TMRM (0.1 μM) were added 5 min prior to the addition of the mitochondria. Ca^2+^ and ADP (1 mM) were added, respectively, 5 min and 10 min after the addition of the mitochondria. All experiments were terminated at 15 min. n = 3 per group. ** *p* < 0.01 and *** *p* < 0.001 vs. Con; ^##^ *p* < 0.01 vs. Ca^2+^.

**Figure 4:**
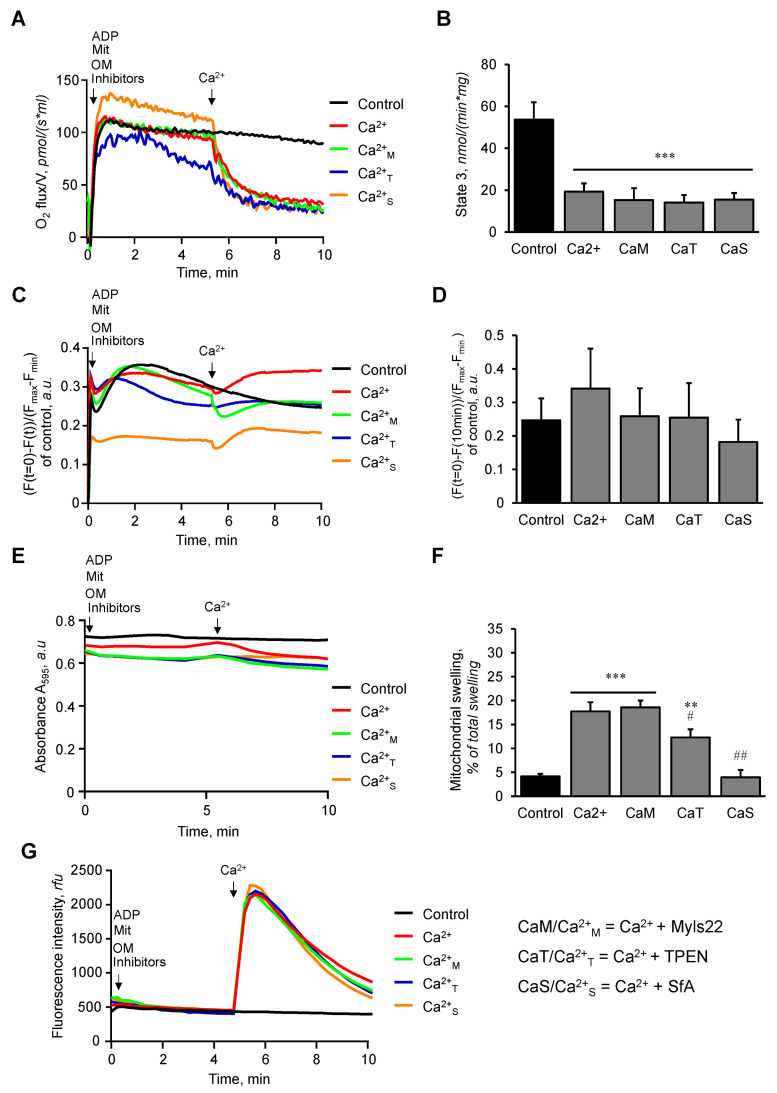
The effects of Myls22 and TPEN on the functional state of mitochondria in the presence of ADP. The effects of Myls22 and TPEN on mitochondrial respiration (**A**,**B**), membrane potential (**C**,**D**), swelling (**E**,**F**), and CRC (**G**) were analyzed in the presence of Ca^2+^ (600 nmol/ml), OM, and ADP. Quantitative data for state 3 respiration rate (**B**), membrane potential (**D**), and mitochondrial swelling (**F**) are presented at 10 min after addition of Ca^2+^. The inhibitors OM, ADP, and TMRM were added 5 min prior to the addition of the mitochondria, and Ca^2+^ was added 5 min after the addition of the mitochondria. All experiments were terminated at 10 min. n = 3 per group. ** *p* < 0.01 and *** *p* < 0.001 vs. Con; ^#^ *p* < 0.05 and ^##^ *p* < 0.01 vs. Ca^2+^.

Next, we evaluated the effects of Myls22 and TPEN on mitochondria exposed to Ca^2+^-induced swelling in the presence of OM (Figure 3) or OM and ADP (Figure 4). We found that Myls22, TPEN, and SfA were not able to prevent the Ca^2+^-induced inhibition of mitochondrial respiration and dissipation of the membrane potential in the presence of OM (no ADP) (Figure 3A–D). Likewise, Myls22 and TPEN had no effects whereas SfA reduced Ca^2+^-induced swelling and increased the CRC of the mitochondria (Figure 3E,F). Interestingly, Myls22 and TPEN significantly reduced mitochondrial respiration (~72% and ~74%) in the presence of OM and ADP (state 3) upon Ca^2+^ addition in comparison to the control group (no Ca^2+^) (Figure 4A,B). However, the mitochondrial membrane potential was sustained after the addition of Ca^2+^ (Figure 4C,D), demonstrating the protective effects of ADP. In the presence of OM and ADP, Ca^2+^-induced mitochondrial swelling was not affected by Myls22. However, both TPEN and SfA (reduced by ~30% and 78%, respectively) had a protective effect and attenuated Ca^2+^-induced mitochondrial swelling (Figure 4E,F). Similarly, both Myls22 and TPEN demonstrated no effects on the mitochondrial CRC, indicating the absence of an mPTP opening under this condition (Figure 4G). Additionally, we performed the same experiments with low Ca^2+^ (300 nmol/mL Ca^2+^) while evaluating mitochondrial respiration and membrane potential. Like high Ca^2+^ concentration, Myls22 and TPEN had no protective effects on mitochondrial respiration and membrane potential at low Ca^2+^ (Figure S1A–D). The results of these experiments demonstrate that TPEN and SfA, but not Mysl22, prevent mPTP-independent mitochondrial swelling under high Ca^2+^ conditions with no protective effects on mitochondrial function.

### 3.3. L-OPA1 Cleavage under Distinct Mitochondrial Energetic and Swelling Conditions

**Figure 5:**
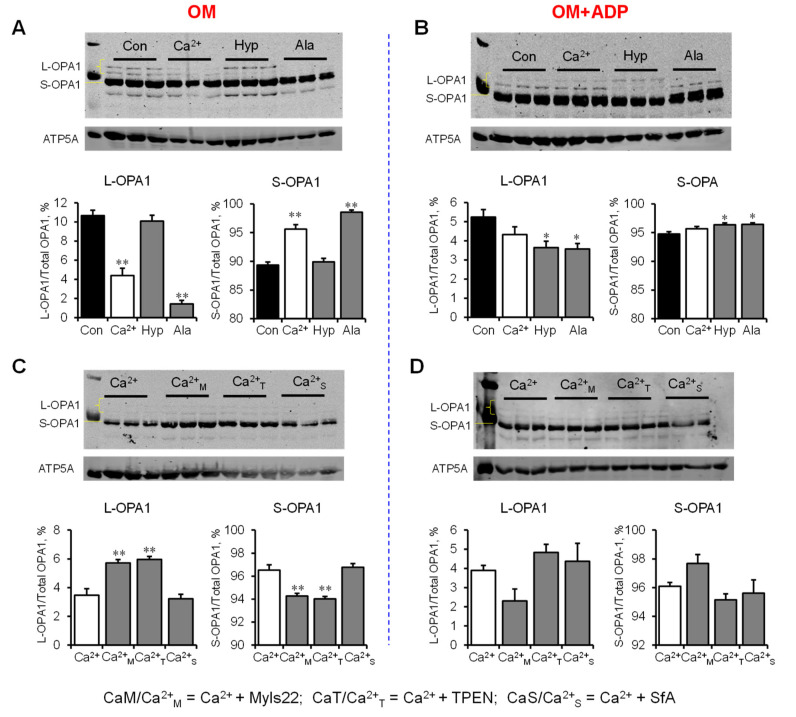
The effects of Myls22 and TPEN on L-OPA1 cleavage under different swelling conditions in mitochondria. L-OPA1 and S-OPA1 protein levels in OM (**A**,**C**) and OM+ADP (**B**,**D**) groups. L-OPA1 cleavage was induced by distinct swelling inducers, including Ca^2+^ (600 nmol/ml), alamethicin (Ala, 10 µM), and hypotonic solution (Hyp) in the presence and absence of Myls22, TPEN, and SfA. L-OPA1 and S-OPA1 were separated using SDS-PAGE and identified by Western blotting using specific OPA1 antibodies. The data obtained from the blots were analyzed using ImageJ software, and the protein levels of L-OPA1 and S-OPA1 were normalized to total OPA1 and expressed as a percentage. ATP5a was used as a loading control. n = 3 per group. * *p* < 0.05, ** *p* < 0.01 vs. Control (Con) for (**A**,**B**) or vs. Ca^2+^ for (**C**,**D**).

**Figure 6:**
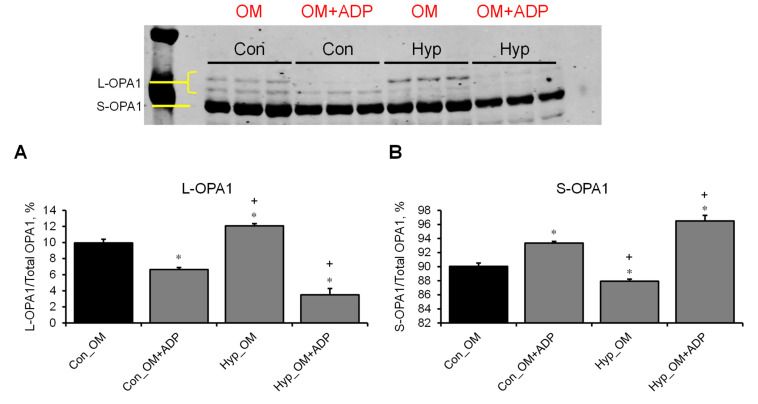
The effects of ADP on L-OPA1 cleavage under hypotonic conditions. The protein levels of L-OPA1 (**A**) and S-OPA1 (**B**) were assessed in OM and OM+ADP groups in isotonic (Con) and hypotonic (Hyp) conditions. L-OPA1 and S-OPA1 were separated using SDS-PAGE and identified by Western blotting using specific OPA1 antibodies. The data obtained from the blots were analyzed using ImageJ software, and the protein levels of L-OPA1 and S-OPA1 were normalized to total OPA1 and expressed as a percentage. n = 3 per group. * *p* < 0.01 vs. Con_OM and ^+^ *p* < 0.01 vs. Con_OM+ADP.

Next, we evaluated L-OPA1 cleavage by analysis of L-OPA1 and S-OPA1 protein levels in the samples from the experiments described in 3.2. We found that mitochondria treated with Ca^2+^ or alamethicin (reduced ~58% and ~86%) in the presence of OM alone (OM group) contained low L-OPA1 levels (Figure 5A). In the presence of OM and ADP (OM+ADP group), Ca^2+^ had no effects, whereas alamethicin or hypotonic medium reduced (approximately 30% and 31%, respectively) L-OPA1 levels (Figure 5B). A hypotonic medium was used to clarify whether an increase in mitochondrial volume can affect the integrity of L-OPA1. As expected, Myls22 and TPEN, but not SfA, attenuated Ca^2+^-induced cleavage of L-OPA1 in the presence of OM as evidenced by high levels of L-OPA1 (approximately 64% and 72% for Myls22 and TPEN, respectively) and low levels of S-OPA1 in these groups compared to the Ca^2+^-treated group used as a control (untreated with Myls22 and TPEN) (Figure 5C). Interestingly, Myls22, TPEN, and SfA had no effects on L-OPA1 levels in mitochondria treated with OM and ADP (OM+ADP group) in the presence of Ca^2+^ (Figure 5D). Next, we compared the protein levels of L-OPA1 and S-OPA1 in the presence of either hypotonic medium or the swelling assay buffer (Figure 6A,B). The results showed that the mitochondria contained less L-OPA1 and high S-OPA1 levels in both the hypotonic medium and the swelling assay buffer in the presence of OM and ADP (OM+ADP group) compared to the OM group. Altogether, these data demonstrate that cleavage of L-OPA1 is stimulated at state 3 respiration but not at state 2, suggesting that L-OPA1 cleavage requires high OXPHOS activity.

## 4. Discussion

Although the role of OPA1 in mitochondrial swelling has been thoroughly investigated, few studies have elaborated on the relationship between OPA1 cleavage and mitochondrial swelling. This study, for the first time, demonstrates a relationship between mitochondrial respiration and L-OPA1 cleavage under physiological conditions. We showed that an increase in mitochondrial respiration leads to more L-OPA1 cleavage, whereas a lower respiratory state is associated with less L-OPA1 cleavage. Next, we demonstrated that Myls22 and TPEN are ineffective in preventing mitochondrial swelling in OM groups even though both agents are effective in partially preventing L-OPA1 cleavage. Moreover, Myls22 and TPEN did not affect the functional state of mitochondria and OPA1 cleavage in the presence of OM and ADP, even though TPEN had some protective effect against mitochondrial swelling. These data suggest that, in addition to its role in fusion, L-OPA1, as well as S-OPA1, can participate in the regulation of the respiratory function of mitochondria.

Using Myls22 and TPEN, we demonstrated that the normal functioning of mitochondria (membrane potential and respiration) requires the structural integrity of the total OPA1 (both L- and S-OPA1). It should be noted that control (untreated) mitochondria isolated from healthy hearts contain a large proportion of S-OPA1, which accounts for ~90% of the total OPA1 (under these experimental conditions). This suggests that S-OPA1 can play a structural and/or functional role in mitochondria. In the present study, a K^+^-based medium was used to replicate cytosolic conditions at 37 °C for 15 min, which better represents a natural setting for evaluating mitochondrial swelling. However, it is important to note that K^+^ induces a background swelling process [25] due to the influx of K^+^ when the mitochondria replenish the lost K^+^ during the isolation procedure. In previous studies, we employed a sucrose-based swelling buffer that lacks basal swelling due to the absence of K^+^, which typically displays an L-OPA1 isoform percentage in the range of 20–25% [15,26,27]. Considering that changes in mitochondrial volume can impact OPA1 processing, we propose that the K^+^-induced swelling could contribute to the observed discrepancy in the OPA1 expression levels.

We investigated the impact of severe stress conditions and distinct respiratory conditions on the reduction in L-OPA1, a protein essential for mitochondrial activity. As shown in Figure 5, under severe stress conditions (Ca^2+^ stress, alamethicin, hypotonic medium) and distinct respiratory conditions (OM, OM+ADP), we observed only a small reduction in L-OPA1. Interestingly, OPA1 GTPase activity is required for mitochondrial activity; nucleoside diphosphate kinase-D (NDPK-D) converts GDP to GTP by utilizing ATP transported through adenine nucleotide translocase (ANT), which can also play a role in modulating mitochondrial swelling [28,29]. Depletion of total OPA1 has shown the development of mitochondrial dysfunction characterized by respiratory defects, lower membrane potential, and IMM swelling [30,31]. Initially, the generation of S-OPA1 due to L-OPA1 cleavage was considered detrimental and with the potential to lead to cell death [8]. Notably, studies with artificial liposomes demonstrated that S-OPA1 resulting from L-OPA1 cleavage retains GTPase activity and dynamin protein functionality [22], suggesting that S-OPA1 can function as a structural IMM protein and interact with different IMM proteins due to its solubility. In favor of this conclusion, recent studies with expression of exclusively L-OPA1 or S-OPA1 in cells showed that S-OPA1 in the absence of L-OPA1 was able to maintain the cristae structure and activity of mitochondrial bioenergetics [3,9]. This suggests that if OPA1 is present in either short or long isoform, the mitochondria can maintain their functional and structural integrity. Using pharmacological inhibitors, we revealed that the transition of mitochondrial respiration from state 2 to state 3 stimulates OPA1 cleavage. Consistent with our data, experiments measuring mitochondrial respiration in tissues overexpressing OPA1 have demonstrated increased mitochondrial complex I respiration compared to the control samples [2]. This observation suggests that the elevated expression of OPA1 can result in the generation of higher levels of S-OPA1 during states of enhanced respiration. Interestingly, there was no difference in complex I mtDNA levels or translation [2]. Moreover, in mice that were exposed to feeding and starvation states, OPA1 could rapidly and reversibly oligomerize in response to changes in energetic demand due to an interaction between the OPA1 and solute carrier 25A (SLC25A) family proteins [32]. However, that study did not evaluate OPA1 cleavage; therefore, new studies are necessary to clarify the potential role of S-OPA1 in OPA1-SLC25A interaction as well as in OPA1 oligomerization that can be affected by shifts in energy demand.

Mitochondrial OPA1 processing occurs through the enzymatic activity of YME1L and OMA1. Under cellular stress, OMA1 activation is demonstrated to cleave L-OPA1 isoforms and lead to mitochondrial fragmentation, which is an underlying factor for the pathogenesis of many diseases such as cardiac ischemia-reperfusion injury [26,33]. However, the ATP-dependent OPA1 protease YME1L cleaves OPA1 without affecting morphology. Genetic studies evaluating OMA1 and YME1L knockout in mouse embryonic fibroblasts (MEFs) showed that mitochondria in the absence of YME1L become fragmented and cannot sustain mitochondrial cristae structure, whereas the depletion of OMA1 does not exhibit these changes [34]. Additionally, YME1L-deficient spinal cord mitochondria exhibit a late onset of respiratory dysfunction [35], and yet another study showed that OMA1-deficient mouse liver mitochondria did not exhibit respiratory dysfunction under a control diet [36]. Overall, these studies suggest that the distinct OPA1 cleavage sites (the S1 and S2 sites) may play different roles toward mitochondrial function, and therefore YME1L and/or OMA1 may have alternative roles independent of the OPA1 processing.

Myls22, a specific inhibitor of OPA1 GTPase activity, has also been shown to suppress mitochondrial fusion [23,37]. Contrary to our expectations, the results of our study did not demonstrate any modulation of Ca^2+^-induced mitochondrial swelling by Myls22. Likewise, TPEN, a zinc-chelator and OMA1 inhibitor [17,38], was not effective in preventing Ca^2+^-induced mitochondrial swelling. It is tempting to speculate that OPA1 oligomerization can be more essential for maintaining the mitochondrial cristae structure than OPA1 cleavage as demonstrated by the genetically modified MEFs, which were able to maintain a moderate cristae structure with only S-OPA1 expression [9]. Moreover, mice starvation experiments have demonstrated that OPA1 oligomerization is necessary to maintain mitochondrial structural integrity under starvation stress [32]. Additionally, OPA1 oligomerization is targeted during BID activation via apoptosis, thereby aiding in the release of cytochrome c and debilitating the structural integrity of the mitochondrial cristae structure [4,39]. The role of OPA1 oligomerization and its relationship to mitochondrial cristae structure and respiration remain to be elucidated in future studies.

Mitochondrial fusion and fission play a central role in maintaining mitochondrial health during physiological conditions [40,41,42]. The interplay between mitochondrial dynamics, mitophagy, and biogenesis is important for maintaining mitochondrial quality control. Changes in the structural remodeling of the IMM play a key role in the adaptation of mitochondria to metabolic/energy demands. The balance between mitochondrial dynamics and energy demand is important for the regulation of mitochondrial bioenergetics [43,44]. In favor of this conclusion, ATP depletion induced by 2-deoxyglucose significantly upregulated fusion proteins (Mfn1 and Mfn2) and reduced the fusion protein Drp1 levels in DLD-1 cells [45]. Similar results were obtained in experiments with oxygen-glucose deprivation [46]. The adult rat heart contains all the fission (Drp1, Fis1) and fusion (Mfn1/2, OPA1) proteins [47]. OPA1 is vital in mediating IMM fusion since it works together with cardiolipin to tether distinct membranes for the fusion process [48]. Furthermore, L-OPA1 and S-OPA1 work together to expedite the membrane tethering process; ideally, a 1:1 ratio of [S-OPA1]/[L-OPA1] provides the most effective fusion conditions for mitochondrial membranes [5]. In turn, studies in MEFs involving non-simultaneous knockout of YME1L and OMA1 as well as double knockout revealed that OPA1 processing is not essential for mitochondrial fusion; however, it may play a stimulatory role in mitochondrial fission [34]. These studies suggest that other factors or mechanisms may be involved in regulating mitochondrial fusion independently of OPA1 processing. Subsequently, many studies have focused on the relationship between mitochondrial fusion and fission toward metabolism [49,50]. However, only a few studies have elucidated the mechanism by which mitochondrial respiration can mediate changes in fusion and fission dynamics. OPA1 cleavage by YME1L, not OMA1, has been shown to result in an OXPHOS-stimulated mitochondrial fusion [51]. Our study demonstrates that alterations in the mitochondrial energetic state led to changes in the S-OPA1/L-OPA1 ratio that may play a causal role in membrane fusion events. Further studies are necessary to clarify the molecular mechanisms underlying the effects of distinct metabolic states on mitochondrial dynamics through OPA1 processing.

## 5. Conclusions

This study provides evidence that L-OPA1 cleavage is associated with mitochondrial respiration, implying that under physiological conditions, L-OPA1 cleavage may favor mitochondrial respiration. Pharmacological inhibition of OPA1 or OMA1 does not affect Ca^2+^-induced mitochondrial swelling, suggesting that the maintenance of cristae structural integrity by OPA1 occurs through an alternate mechanism(s). OPA1 oligomerization seems to play an important role in maintaining mitochondrial structural and functional integrity during stress conditions.

### Limitations

This section aims to acknowledge the limitations of our study and provide a balanced perspective on the findings. Firstly, accurately quantifying faint L-OPA1 bands compared to stronger S-OPA1 bands was challenging and may have introduced uncertainties into the findings. Specifically, as part of our research, we conducted a comparative analysis of the effects of various compounds/factors on L-OPA1 and S-OPA1 under two distinct conditions: (i) preservation of mitochondria in a sucrose buffer on ice and (ii) exposure to a K^+^-based swelling assay buffer at 37 °C. We observed an ~15% reduction in L-OPA1 isoforms in the K^+^-based buffer, which caused the L-OPA1 bands to become fainter. Presently, our investigation aims to elucidate the underlying reasons for this observed phenomenon, with particular emphasis on discerning the potential role of passive K^+^-dependent swelling as a plausible contributing factor. Secondly, the focus on measuring changes in mitochondrial volume using the mitochondrial swelling assay might not fully capture all the factors affecting mitochondrial behavior. Complementing this approach with other assays that assess mitochondrial function or morphology could provide a more comprehensive view of mitochondrial dynamics. The absence of Na^+^ in the assay was intended to prevent confounding effects, but it may also limit the generalizability of the results. Another limitation lies in the changes made to the experimental environment, such as using a K^+^-based buffer and incubation at 37 °C, which might alter the OPA1 values. These environmental modifications may introduce variations into the experimental conditions, influencing the behavior of the OPA1 and potentially affecting the accuracy and comparability of the results. Despite these limitations, the study provides valuable insights into mitochondrial dynamics and mPTP regulation, and future research could address these issues to further enhance understanding.

## Data Availability

The data that support the findings of this study are available from the corresponding author, upon reasonable request.

## Supplementary Materials

The following supporting information can be downloaded at: https://www.mdpi.com/article/10.3390/cells12162017/s1.
