# Supplementary material for: The Role of Swelling in the Regulation of OPA1-Mediated Mitochondrial Function in the Heart In Vitro
Source: Cells. 2023 Aug 8;12(16):2017. doi: 10.3390/cells12162017 (PMC10453793; doi:10.3390/cells12162017)
Supplement: Supplementary file 1 [file cells-12-02017-s001.zip › cells-2485044-supplementary.pdf]

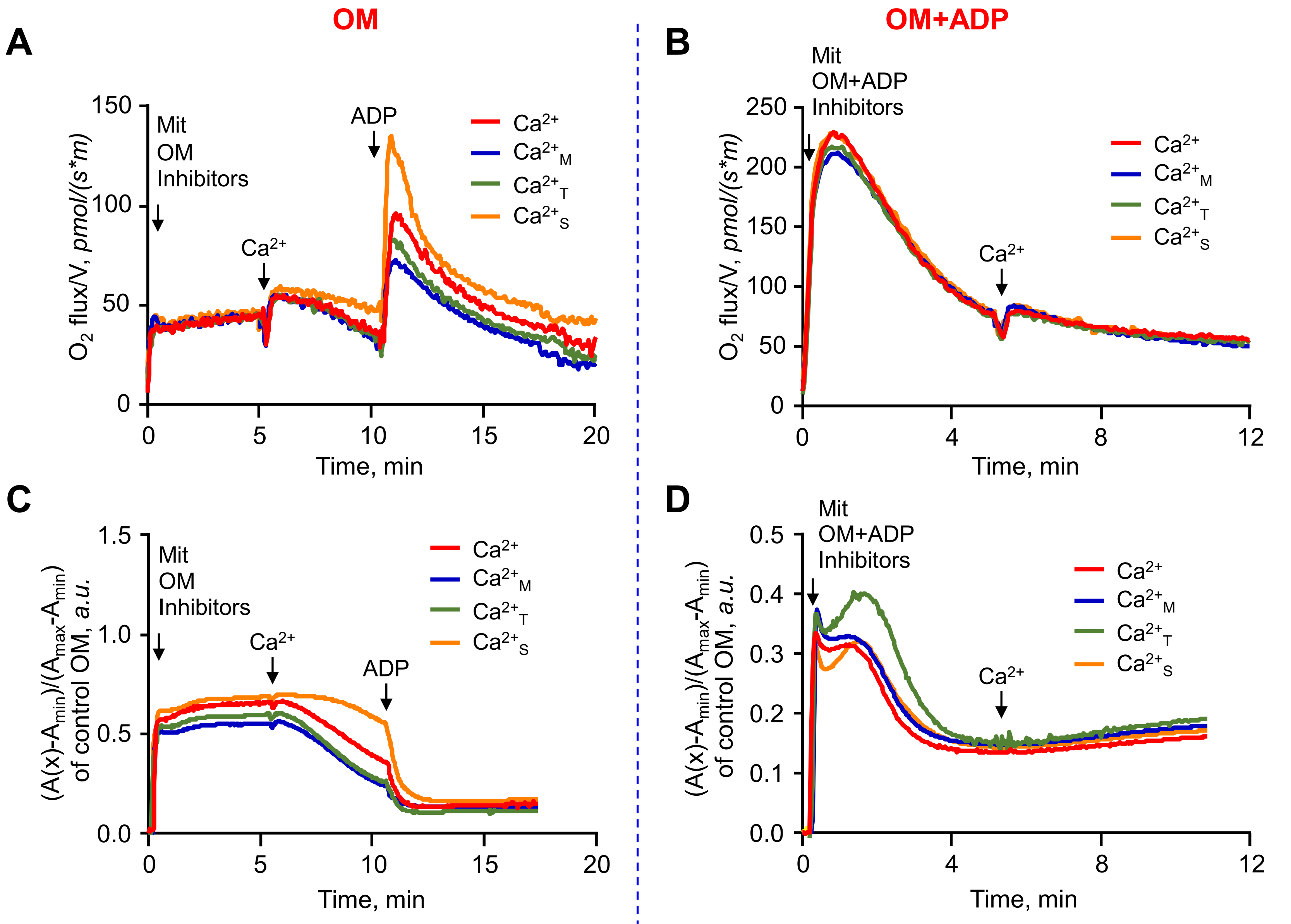

Ca^{2+}_M = Ca^{2+} + Myls22; Ca^{2+}_T = Ca^{2+} + TPEN; Ca^{2+}_S = Ca^{2+} + SfA

Figure S1: The effects of mitochondrial swelling induced by low Ca^{2+} on mitochondrial respiration and membrane potential in the presence and absence of Myls22 and TPEN. Mitochondrial respiration (**A**,**B**) and membrane potential (**C**,**D**) was evaluated in OM and OM+ADP groups in the presence and absence of Ca^{2+} (300 nmol/ml). n=3 per group.
